# Perceptions of physiotherapists regarding challenges experienced by patients with Deafness in accessing rehabilitative care

**DOI:** 10.4102/sajp.v81i1.2241

**Published:** 2025-10-11

**Authors:** Meluleki S. Thethwayo, Olivia Baloyi

**Affiliations:** 1 Discipline of Physiotherapy, College of Health Sciences, University of KwaZulu-Natal, Durban, South Africa; 2 School of Nursing and Public Health, College of Health Sciences, University of KwaZulu-Natal, Durban, South Africa; 3 Department of Nursing, Faculty of Health Sciences, Walter Sisulu University, Mthatha, South Africa

**Keywords:** Patients with deafness, physiotherapists, rehabilitative care, physiotherapy, communication barriers

## Abstract

**Background:**

Hearing impairment, including deafness, affects approximately 5% of the global population, with the majority residing in low- and middle-income countries (LMICs) such as South Africa. Despite the constitutional right to healthcare for all, patients with deafness face persistent health inequalities because of communication barriers with healthcare professionals. Physiotherapists are often unprepared to provide care for patients with deafness, leading to a breakdown in communication and poor healthcare outcomes.

**Objectives:**

This study explored physiotherapists’ perceptions regarding the challenges faced by patients with deafness in accessing rehabilitative care in South Africa.

**Method:**

A qualitative research approach was used, with social constructivism as the guiding paradigm. In-depth individual interviews were conducted with 18 purposively selected physiotherapists across three public hospitals in KwaZulu-Natal, South Africa. Data were analysed using Elo and Kyngäs content analysis framework, which involves a systematic three-phase approach.

**Results:**

The study identified five main categories: (1) access to rehabilitative care, (2) communication barriers, (3) disability stigma and cultural barriers, (4) healthcare system challenges and (5) future aspirations.

**Conclusion:**

The findings highlight the need for improved communication strategies, sign language training and a more inclusive approach within the healthcare system to enhance access to rehabilitative care for patients with deafness. The study underscores the importance of addressing systemic and individual factors to provide equitable healthcare to all patients, including those with hearing impairments.

**Clinical implications:**

The study underscores the urgent need for physiotherapists to receive sign language and cultural competency training to improve communication and healthcare delivery for patients with deafness, ensuring equitable access to rehabilitative care.

## Introduction

The World Health Organization (WHO 2023) projected that approximately 5% of the world’s population lives with some form of hearing impairment, which includes deafness. About 80% of this population is found in low- and middle-income countries (LMICs), with South Africa (SA) being no exception (WHO 2023). According to the Constitution of the Republic of South Africa 108 (1996), access to healthcare is a legal right for people, including people with deafness. However, despite this considerable statement, patients with deafness are still experiencing persistent health inequalities (Baratedi et al. 2021; Snögren et al., 2023). Remaining at the forefront of the challenge is the communication barrier that exists between patients with deafness and hearing healthcare professionals (Baratedi et al. 2021; Gichane et al. 2017; Snogren et al. 2023). These communication challenges are rooted in the practices of discrimination, stigmatisation, the lack of awareness about the disability and inadequate preparedness and training of healthcare professionals (including physiotherapists) to manage patients with deafness (Baratedi et al. 2021; Snögren et al. 2023).

The lack of trained and qualified sign language interpreters further contributes to the existing communication barrier between patients with deafness and healthcare professionals within the South African healthcare sector (Baloyi, Walters & Jarvis 2023; Baratedi et al. 2021). While the provision of qualified sign language interpreters is viewed as a feasible approach to mitigate the challenge of communication barriers between patients with deafness and healthcare professionals (Akeely et al. 2022), the issue of patient confidentiality remains a concern (Baloyi et al. 2023; Baratedi et al. 2021; Senne 2016). The use of sign language interpreters is not a concern with medical confidentiality only, but it can be associated with the omission of crucial medical information during the interpretation, which can further result in unnecessary medico-legal cases (Abou-Abdallah et al., 2021; Akeely et al. 2022; Sporek et al. 2014). The provision of quality healthcare relies on good communication between the patient and the healthcare professionals (Agaronnik et al. 2019). However, patients with deafness are deprived of this privilege, which ultimately results in them receiving poor healthcare services (Baloyi et al. 2023; Haricharan et al. 2013). The introduction of the South African sign language in all health science professional degrees is recognised as a feasible solution to produce professionals who can provide inclusive healthcare services to all, including patients with deafness (Akeely et al. 2022; Baloyi et al. 2023; Baratedi et al. 2021; Snogren et al. 2023).

Physiotherapists are integral members of the multidisciplinary team who provide rehabilitative care to patients with musculoskeletal, neurological, orthopaedic, paediatric and respiratory conditions using various treatment techniques to prevent further complications (Fullen et al. 2023; Kim et al. 2022; Pedersini et al. 2021). In addition, these healthcare professionals provide individual patient education about managing and preventing chronic diseases, which often involves prescribing home exercise programmes to ensure the continuity of patient care. This means that effective communication between physiotherapists (like any other healthcare professional) and the patients (including patients with deafness) receiving rehabilitative care is an essential element to producing the best patient outcomes (Agaronnik et al. 2019; Haricharan et al. 2013; Kwame & Petrucha 2020). Through effective communication, physiotherapists can establish a good rapport with patients for whom they are caring, as thoughts and emotions are shared during the process, which makes it easy to develop the best treatment plan (Ayala-Hernandez et al. 2021; Snögren et al. 2023). However, patients with deafness can experience challenges in benefiting from the medical information provided by healthcare professionals, including physiotherapists, during the communication process, which ultimately results in poor patient outcomes (Baloyi et al. 2023; Snögren et al. 2023). As far as the researchers are aware, there is little data regarding the difficulties faced by deaf patients in obtaining rehabilitation services in South Africa. This study purposefully examined the problem from the viewpoint of physiotherapists, even though the research gap is obviously focused on the experiences of patients with deafness. As important rehabilitative care providers, physiotherapists frequently work directly with patients with deafness, often in difficult situations. Their perspectives provide important information about the communication, systemic and training-related obstacles affecting patients’ care access. This study provides a fundamental understanding of the institutional and professional limitations affecting providing services to patients with deafness by concentrating on the provider perspective. Future studies employing inclusive and accessible methodologies that directly involve people with deafness may benefit from these findings.

## Research methods and design

### Research approach and design

In agreement with Charmaz (2017), the authors of this paper believe that knowledge is co-constructed through social interactions, experiences and contexts. Hence, social constructivism was chosen as the guiding paradigm for this study. In addition, a qualitative research approach was employed to capture rich, detailed insights into experiences and individual perceptions of physiotherapists regarding challenges experienced by patients with deafness in accessing rehabilitative care.

### Study setting

The study was conducted in three public hospitals in the KwaZulu-Natal (KZN) province, South Africa. The first was a tertiary hospital located on the north coast of KZN, serving the rural communities of King Cetshwayo, Umkhanyakude and Zululand districts. The other two hospitals were Level 2 regional hospitals: one based in eThekwini (serving the eThekwini district) and the other on the south coast of KZN (serving the Ugu district). These hospitals provide rehabilitation services across the mentioned districts. These hospital settings were selected as they serve the rural and peri-urban communities in KZN, which typify the level of public-sector rehabilitative care accessed by persons with disabilities in rural settings (Magaqa Ariana & Polack 2021; Vergunst et al. 2017).

### Study population, sampling and sample size

A non-probability, purposive sampling method was used to select all (*N* = 18) permanently employed physiotherapists across the three study sites. The authors recruited the participants face to face at the study sites. The inclusion criteria were that participants must be registered physiotherapists with the Health Professions Council of South Africa (HPCSA), employed at one of the study sites, and voluntarily willing and available to participate.

### Data collection

Data were collected through in-depth individual interviews conducted by the principal investigator (M.S.T.). The data collection period spanned from 01 November 2024 to 15 January 2025. Participants were asked a central open-ended question using an interview guide: ‘What are your perceptions regarding the challenges experienced by patients with deafness in accessing rehabilitative care?’ To encourage further elaboration, probing questions were used, such as: ‘Can you explain further?’ ‘What do you mean?’ and ‘Is there anything else you would like to add?’ These questions helped to gain a deeper understanding of the participants’ experiences. Furthermore, the researcher collected demographic data to obtain the participants’ working experience, gender, age, number of patients with deafness they have managed as a physiotherapist, and the undergraduate institution attended. Interviews were conducted in a quiet, unoccupied room in the study sites, ensuring an environment free from interruptions. Each interview lasted between 45 min and 1 h and was audio-recorded for accuracy. The recordings were then transcribed verbatim. All interviews were conducted in English. Data saturation was determined during the coding and analysis process. After the 12th interview, no new themes, codes or categories emerged. The information provided was largely repetitive and confirmed patterns already identified. To strengthen the credibility of the findings and ensure data richness, the researcher conducted interviews with all 18 physiotherapists at the study sites.

### Data analysis

Data analysis was conducted manually by both researchers (M.S.T. and O.B.) using Elo and Kyngäs’ (2008) content analysis framework, which involves a systematic three-phase process: preparation, organising and reporting. The principal investigator (M.S.T.) led the preparation phase by transcribing the 18 individual interviews verbatim. The transcripts were then shared with O.B., who cross-checked the transcriptions against the audio recordings. This process allowed both researchers to become deeply immersed in the data through repeated listening and re-reading of the transcripts, ensuring a thorough understanding of the content. In the organising phase, both researchers independently developed categories and sub-categories. Through further discussion and collaboration, they finalised 5 categories and 12 sub-categories. The final phase, the reporting phase, involved describing the categories in detail (Elo & Kyngäs 2008).

### Rigour

Lincoln and Guba’s (1985) criteria were followed to enhance trustworthiness. An interview guide was used during individual interviews to generate rich, detailed information to ensure transferability. Member checking was employed to verify the accuracy of the data, ensuring it was a true reflection of participants’ input. The transcripts were shared with participants for verification, allowing them to confirm the data and the inferences drawn by the researcher from their responses. Participants were also allowed to make corrections if necessary. To ensure confirmability, the researchers maintained an audit trail, which included audio recording, verbatim transcription of individual interviews and written field notes. Data collection continued until saturation and redundancy were achieved, and both researchers performed independent coding. A comprehensive and detailed description of the research methodology was provided to ensure dependability.

### Ethical considerations

Ethical clearance (reference number: HSSREC/00007678/2024) was obtained from the University of KwaZulu-Natal, Humanities and Social Sciences Research Ethics Committee. In addition, gatekeeper permissions were secured from the study sites and the KwaZulu-Natal Department of Health. The Principal Investigator (M.S.T.) obtained verbal and written consent from all participants. Participants signed consent forms to confirm their participation in the study and their agreement to be audio recorded. Confidentiality and anonymity were strictly maintained throughout the study, with participants being referred to by their chosen pseudonyms.

## Results

Eighteen physiotherapists (*n* = 3 [16, 7%] males; *n* = 15 [83, 3%] females), ranging in age from 23 to 59 years (mean = 33.6 years), participated in this study (Table 1). The participants had at least 1 year of experience and worked in physiotherapy departments at the study sites.

**TABLE 1:** Participant demographics (*N* = 18).

Participant number	Age (years)	Gender	Years of experience
P1	32	Female	8
P2	27	Female	5
P3	41	Female	17
P4	30	Female	7
P5	33	Female	8
P6	30	Female	7
P7	59	Male	26
P8	40	Female	17
P9	48	Female	15
P10	27	Female	5
P11	27	Female	5
P12	34	Female	10
P13	23	Female	1
P14	28	Female	5
P15	28	Female	5
P16	28	Male	6
P17	27	Male	5
P18	44	Female	19

### Study’s findings

Five categories and 12 sub-categories supported by verbatim transcripts emerged from the data following content analysis (Table 2).

**TABLE 2:** Categories and sub-categories that emerged from collected data.

Categories	Sub-categories
Access to rehabilitative care	Lack of knowledge about physiotherapy services Financial burden
Communication barriers	Poor understanding of basic sign language Relying on family members for interpretations Lack of trained sign language interpreters
Disability stigma and cultural barriers	Negative attitudes of the physiotherapists Cultural beliefs and practices surrounding disability
Healthcare system challenges	Ill-equipped healthcare professionals Time constraints coupled with a shortage of medical personnel
Future aspiration	Integration of sign language in healthcare education Ongoing professional development and in-service training Development of disability policy for patients with deafness

### Category 1: Access to rehabilitative care

Under this category, the participants highlighted factors that hinder access to rehabilitation services for patients with deafness seeking physiotherapy services. These were related to awareness and affordability, as outlined in the following sub-categories:

#### Sub-category 1.1: Lack of knowledge about physiotherapy services

The participants indicated that oftentimes, patients with deafness are not aware of the available physiotherapy rehabilitative services accessible to them since the majority of these services are not tailored to meet their specific communication needs. According to them, this lack of awareness results in either inpatient delays or avoidance of necessary rehabilitation, leading to worsened health conditions that could have been addressed with early intervention. The following extracts summarise how participants verbalised this particular study finding:

‘I have seen not more than two deaf patients, and I think sometimes it’s a lack of information. You know, so there might be a lack of information for caregivers about physiotherapy, and knowing the indications of when a patient needs physiotherapy. Some don’t know about us, especially those who are from deep rural areas and face communication challenges.’ (P1)

‘If no one has ever shared that information with you about physio, how are you going to know unless you meet someone?’ (P18)

#### Sub-category 1.2: Financial burden

Another important factor mentioned by the participants was the financial burden. The participants alluded to the financial resource disparities between different segments of the population with deafness, leading to unequal access to rehabilitative care. Following are some of the verbatim comments from the participants:

‘So, they have to take a tax and get someone into a tax, then pay for two people coming, and that’s a lot of money.’ (P3)

‘Or the learner, it was transport issues because they had to book transport at school to come for rehab. Hence, they came only once; they didn’t complete their rehab follow-up sessions.’ (P18)

### Category 2: Communication barriers

Communication barriers emerged as one of the overarching categories impacting negatively on patients with deafness seeking rehabilitative services. The study participants alluded to the challenges around communication difficulties between them and patients with deafness during the consultation. These are further elaborated in the following sub-categories.

#### Sub-category 2.1: Poor understanding of basic sign language

The study participants mentioned their limited proficiency in sign language, which often created significant barriers in their communication with patients with deafness seeking rehabilitation services, as illustrated in the following extracts:

‘I think it’s the communication more than anything because with physio, we give instructions, a lot of instructions, and we expect them to follow those instructions. So, most of us are not trained to communicate with these people, we don’t know sign language at all.’ (P1)

‘It’s communication. Most of them are using sign language. So, with my patient, the only thing I was doing to get answers was to point out that I didn’t know how to communicate with that patient. It is difficult for a patient to give a full explanation of their pain to us because we have to ask and get answers, so it becomes difficult to get a history from a patient to get their expectations.’ (P6)

#### Sub-category 2.2: Relying on family members for interpretations

The data sources further mentioned that patients with deafness rely on their family members for interpretation purposes during rehabilitation. Although the data sources acknowledged and appreciated the family support for their significant other, they did mention some challenges. These were not limited to family members’ limitations in their understanding of medical terminology and their capability and accuracy in conveying complicated information, notwithstanding the possibility of patients’ breach of confidentiality. Some participants had this to say:

‘… they always must have somebody accompanying them so that they can be heard and understood, and unfortunately, this affects the medical information confidentiality of the patient, but it is the only easy way at the moment.’ (P3)

‘It wasn’t me treating the patient, but it was me treating the patient via the relative, it was not direct, and I don’t think we had good enough experience that we were able to build a rapport, and I never saw her again, it was just a bypass session.’ (P5)

‘They [*patients with deafness*] bring their family member who assists in interpreting the information shared during the session. But to be honest, I don’t trust a person if they translate my instruction exactly to the patient without missing anything, as I don’t understand sign language; however, there is nothing I can do.’ (P18)

#### Sub-category 2.3: Lack of trained sign language interpreters

The lack of trained sign language interpreters created a critical gap, as per the data sources. This resulted in patients with deafness not having access to correct and timely interpretation, leading to delays in treatment and misunderstandings of medical advice during rehabilitation. The following extracts evidence this:

‘I’ve been to the awareness and none of that awareness that has interpreters when we go to the community to do awareness, they will announce on the road with the speaker on this day MEC [*Member of Executive Council*] of health is coming with all the rehabilitative services if you got issues that require physiotherapy but if there are no sin language interpreters’ deaf people are excluded automatically.’ (P18)

‘There are no trained and qualified sign language interpreters either in the hospital to help us, so we end up relying on family members, and we don’t know if they pass exact information to these patients.’ (P17)

### Category 3: Disability stigma and cultural barriers

Participants’ responses revealed the significant role stigma and cultural barriers play in shaping attitudes towards individuals with disabilities and their access to rehabilitative care. The following sub-categories emerged from this category, (1) the negative attitudes of physiotherapists and (2) the cultural beliefs and practices surrounding disability.

#### Sub-category 3.1: Negative attitudes of the physiotherapists

Physiotherapists interviewed in this study acknowledged their biases and various attitudes towards patients with deafness seeking rehabilitation physiotherapy services. According to them, they often treated patients with deafness differently. This included involuntarily offering less detailed information about treatment plans, not attending to all aspects of rehabilitation or failing to include patients with deafness in decision-making about their care. The following extracts attest to this:

‘Some of us have a stigma toward these patients. We are impatient when dealing with them. Sometimes give them negative attitudes and we look at them in a strange way when they come for rehab as if they are not complete human beings, as we don’t involve them directly, even during the session, it is only via a family member.’ (P16)

‘There are higher chances of deaf people experiencing negative attitudes from healthcare professionals, especially as deafness is not a physical disability. For example, if you call a patient to come and the patient does not come because the patient did not hear you, you will get irritated. And eventually, you display a negative attitude to the patient.’ (P18)

#### Sub-category 3.2: Cultural beliefs and practices surrounding disability

Aligned with this sub-category, the physiotherapists in the study shared their views on how cultural beliefs and practices shaped how they interacted with patients with deafness during rehabilitation. According to them, some cultures promote self-reliance, discouraging patients with deafness from seeking external help, thus avoiding exposing personal struggles with the disability itself. According to the data sources, the latter may prevent patients with deafness from seeking rehabilitative services because they believe they should demonstrate independence. The following excerpts depict participants’ views in this context:

‘Other families hide those patients and just take care of them at home. I think it’s a stigma, more than anything, in rural areas because there is a cultural belief that if someone gave birth to a disabled person, there’s a problem, maybe the person is cursed.’ (P1)

‘So, most people in rural areas are stigmatising the disability, so families end up keeping them home and just go to the hospital only if they need a grant.’ (P8)

### Category 4: Healthcare system challenges

Aligned with this category, physiotherapists explained their challenges within the healthcare system, which negatively affected their ability to rehabilitate patients with deafness. The following sub-categories delve deeper into these healthcare system challenges.

#### Sub-category 4.1: Ill-equipped healthcare professionals

The physiotherapists perceived themselves as not fully equipped to adapt rehabilitation techniques to accommodate the needs of patients with deafness. They mentioned that modifying exercises or rehabilitation plans was hard to ensure that patients with deafness could fully understand and participate in the process. The following quotations give evidence of the physiotherapists’ struggles:

‘We can never know when a patient is going to come, but we are not ready to see that patient one-on-one.’ (P3)

‘So even in our training, we don’t learn enough about dealing with deaf patients.’ (P7)

#### Sub-category 4.2: Time constraints coupled with a shortage of medical personnel

A shortage of physiotherapists, coupled with time constraints, emerged in this study as healthcare system challenges affecting the provision of rehabilitative services to patients with deafness. The following verbatim transcripts from physiotherapists confirm this:

‘Sometimes we end up using things to write down what you are saying and they take a lot of time whereas if you know sign language just like we talking now, we get the message across so quickly, but if we were to start writing that, it’s going to take forever and that wastes our time, especially as we are short-staffed.’ (P3)

‘It is frustrating, I don’t want to lie because instead of seeing that [*patient with deafness*] patient for an hour, you may end up spending 2 hours because everything you want to say, you have to write it down. It is a struggle and frustrating, especially as we are short-staffed.’ (P18)

### Category 5: Future aspiration

Participants offered insightful ideas for future tactics that might tackle current issues in the context of improving the rehabilitative care experience for patients with deafness. These suggestions aimed to ensure that medical personnel were more equipped to interact with patients and deliver high-quality care. The following sub-categories summarise this.

#### Sub-category 5.1: Integration of sign language in healthcare education

To guarantee that medical personnel are prepared to interact with patients with deafness efficiently, participants underlined the significance of integrating sign language into the undergraduate curriculum for all health science degrees. The following excerpts depict participants’ views in this context:

‘I think the first thing that must be introduced is sign language, like it’s an official language and we’re dealing with people living with disabilities, which includes people with deafness, so if it can be compulsory from varsity …’ (P1)

‘Yes, I think it [*sign language*] can be included in undergraduate training just like non-Zulu speaking students are required to learn isiZulu.’ (P3)

#### Sub-category 5.2: Ongoing professional development and in-service training

Participants suggested the adoption of ongoing in-service training and workshops to improve healthcare professionals’ abilities to manage patients with deafness and overcome communication barriers, as shown in the following excerpts:

‘I think in-service training can also help in the hospital settings, just to make sure that everyone is still good, cause if you’re not using something, it’s easy to lose it and don’t always have deaf patients, so we might lose it.’ (P1)

‘We could get in-service training for sign language, it needs to be continuous because you never know when the deaf patient will peach.’ (P3)

#### Sub-category 5.3: Development of disability policy for patients with deafness

Furthermore, the development of disability policy that will address the needs of patients with deafness in the healthcare sector was also recommended by the participating physiotherapists to accentuate the gap in communication breakdown between physiotherapists and patients with deafness. The following illustrative quotes reflect what they said:

‘I hope it will hit some nerves with policymakers so they will include this population [*patients with deafness*] in the policy.’ (P4)

‘For me, honestly speaking, there is a need for policies that speak about that community [*patients with deafness*] directly.’ (P18)

## Discussion

Equal access to universal health is a right for all people, including patients with deafness, and this holds considerable meaning in countries such as South Africa, where human rights are promoted (Constitution of the Republic of South Africa 1996). However, persons with disabilities, particularly in rural settings, are still experiencing inequalities in accessing healthcare services, including rehabilitative care (Magaqa et al. 2021; Vergunst et al. 2017). The selected study settings typify the level of public-sector rehabilitative care accessed by persons with disabilities in rural settings. The article explored physiotherapists’ perceptions about the experiences of patients with deafness in accessing rehabilitative care.

The years of working experience of the participants in this study varied widely; interestingly enough, their perceptions about the experiences of patients with deafness accessing rehabilitative care were largely consistent. Lack of knowledge of health-related information because of poor level of education and communication barriers among patients with deafness limits this group from accessing healthcare services equally as their peers without hearing disabilities (Appiah et al. 2018; Baratedi et al. 2021; Haricharan et al. 2017; Sithinyiwe & Ngonidzashe 2016). In our study, most physiotherapists perceived that poor knowledge about physiotherapy services and illiteracy predispose patients with deafness to rehabilitative care inequalities. Hence, a patient with deafness has to spend more money when accessing rehabilitative care, as they often need family members to convey the information between them and the treating therapist during consultation. This is of concern, as most of the persons with disability are said to be unemployed; they only rely on disability grants (Appiah et al. 2018; Baratedi et al. 2021), which further affects their rehabilitative care-seeking behaviours.

Most healthcare professionals, including physiotherapists, do not understand the sign language often used by patients with deafness (Appiah et al. 2018; Baratedi et al. 2021; Snögren et al. 2023). Similar findings were found in our study, as all physiotherapists admitted that they do not understand the sign language used by patients with deafness when accessing rehabilitative care. The work of Kwame and Petrucha (2020) and Agaronnik et al. (2019) supports effective communication between healthcare professionals and patients with deafness as an essential element for the provision of quality care, as feelings and thoughts are shared during medical history taking to provide the best treatment goals. However, this was lacking in our study as all physiotherapists alluded that establishing a good rapport between them and patients with deafness was challenging because of communication barriers. Physiotherapists had to rely on family members to convey the message between them and patients with deafness. This concerns the level of rehabilitative care afforded to patients with deafness in these institutions. As the inclusion of the third person for interpretations during the consultation is associated with concerns of patient medical information confidentiality and poor history taking, which ultimately results in misdiagnosing the patients and unnecessary medico-legal cases (Abou-Abdallah et al. 2021; Akeely et al. 2022; Baloyi et al. 2023; Baratedi et al. 2021). Interestingly, participating physiotherapists were aware of the issues involving a family member during the consultation. However, this was said to be the only convenient way to render rehabilitative care to patients with deafness in our study.

A lack of trained and qualified sign language interpreters within the public healthcare sector further complicated the communication challenges between physiotherapists and patients with deafness in our study (Baloyi et al. 2023). On the contrary, Appiah and colleagues (2018) stated that using sign language interpreters makes patients with deafness develop mistrust and lose independence during the consultation, as they do not know if their health concerns are adequately communicated without missing some crucial medical information. Nevertheless, Baratedi et al. (2021) still believe that the lack of sign language interpreters exposes patients with deafness to poor healthcare, especially when healthcare professionals arbitrarily conclude the health status of a patient with deafness. This concern is echoed in international recommendations.

The World Federation of the Deaf (WFD) emphasises that access to qualified sign language interpreters in healthcare is a fundamental human right and critical to preventing miscommunication and mistrust. In the South African context, the recent recognition of South African Sign Language (SASL) as the 12th official language (2023) reinforces the need to integrate SASL interpreters into health services to ensure equity, inclusivity, and the protection of the rights of patients with deafness.

In their study, Snögren et al. (2023) and Appiah et al. (2018) found that the negative attitude of healthcare professionals towards patients with deafness prohibited this vulnerable group from accessing equitable healthcare compared to their peers without hearing disabilities. The authors further stated that in some healthcare facilities, healthcare professionals prioritise patients without hearing disabilities over patients with deafness, and they overlook the concerns of this vulnerable group. This is congruent with the findings of our study, as the physiotherapists admitted to being impatient when dealing with patients with deafness during the consultation, and they further alluded that dealing with this group is time-consuming. The challenge of managing patients with deafness is not unique to the South African context. In a study done in the United Kingdom, doctors were uncomfortable providing healthcare to patients with deafness (Barnett, Koul & Coppola 2011). These behaviours are of concern as they are misaligned with Article 25 of the United Nations Convention on the Rights of Persons with Disabilities, which declares that healthcare professionals should not deny equal access to healthcare services to an individual based on disability grounds (United Nations 2006). Masoga and Maoto (2021) state that in some cultures, disability is still perceived to be associated with a curse or disgrace. Similar sentiments were echoed in our study by one of the physiotherapists who alluded that some families hide patients with deafness at home as they believe that this vulnerable group is cursed.

On the other hand, all physiotherapists explicitly admitted that they are not well trained and prepared to provide rehabilitative care to patients with deafness, as the inclusion of sign language was not incorporated in their undergraduate curriculum (Baratedi et al. 2021; Gichane et al. 2017; Snogren et al. 2023). This was regardless of the institutions that our participating physiotherapists attended for their undergraduate training. The shortage of medical personnel, coupled with time constraints in our study (Thethwayo et al. 2024), further complicated the provision of rehabilitative care to patients with deafness. As some physiotherapists alluded, sometimes they need to convey information to patients with deafness by writing it down, which consumes a lot of time when managing this vulnerable group. At the same time, the available human resources in these institutions need to be optimised at the best level because of the dynamics of the existing working environment. However, this does not give physiotherapists the right to be impatient when providing rehabilitative care to patients with deafness. To address these challenges, physiotherapists could receive regular in-service training on deaf awareness and patient rights. At the same time, health sciences curricula can integrate brief sessions within existing modules rather than introducing new courses. In addition, the use of SASL interpreters, remote interpreting services or visual communication aids may serve as practical alternatives to improve communication with patients.

In line with Snögren et al.’ s (2023) and Baloyi et al.’s (2023), all physiotherapists in our study recommended that SASL be introduced at the undergraduate level across all health science professional degrees for future clinical practice. This will assist in producing healthcare professionals who possess the core competencies required to manage patients with deafness in clinical practice. In addition, our findings highlight the importance of addressing underlying prejudices and promoting self-awareness and positive attitudes towards disability, as these are integral to delivering equitable and respectful care.

South Africa has made strides in developing disability policies; however, the currently existing disability policies are too vague and fall short of spelling out how to address diverse disability issues (Mutanga & Walker 2017). The National Development Plan (NDP 2012) declared that there is no one-size-fits-all approach to disability matters, as it acknowledges the diversity around disability. In alignment with this declaration, our participants further recommended that disability policymakers develop a disability policy that will speak directly to the needs of patients with deafness in the southern African healthcare sector. For example, South Africa has implemented policies to support the needs of persons with disabilities, such as ensuring access to braille textbooks for visually impaired learners and creating Deaf-friendly work environments, demonstrating that targeted disability policies are feasible and impactful. Furthermore, ongoing in-service training for staff within the health sector was regarded as a sustainable approach to address disability matters. These recommendations are sound and plausible in ensuring the attainment of the goals of universal health coverage by 2030.

### Limitations

The study was conducted in three healthcare facilities in one province. Therefore, the generalisation of the study findings should be applied with caution. However, future researchers may use the study findings as a baseline. The interview guide was peer-reviewed for suitability, but the study was not piloted to ensure accuracy with the data collection process.

### Recommendations

To improve access to rehabilitative care for patients with deafness, healthcare systems could feasibly incorporate basic sign language training into existing professional curricula, provide targeted in-service training for practitioners and expand the availability of trained sign language interpreters, using cost-effective or technology-assisted solutions where necessary. Public awareness campaigns should inform the population with deafness about available services, while addressing financial barriers through subsidies or transport assistance. In addition, inclusive disability policies tailored to the needs of patients with deafness, along with culturally sensitive education to combat stigma, will help ensure equitable and effective rehabilitation services. These steps will foster a more inclusive healthcare environment for individuals with deafness.

## Conclusion

This study highlights significant barriers faced by patients with deafness in accessing rehabilitative care, particularly in the context of physiotherapy services. Communication challenges, including the lack of sign language proficiency among healthcare professionals and the reliance on family members as interpreters, are major hindrances to effective treatment. In addition, financial constraints and cultural stigma surrounding disability further exacerbate the difficulty patients with deafness face in seeking care. Physiotherapists acknowledged their limitations in managing patients with deafness, citing insufficient training and the absence of dedicated resources such as trained sign language interpreters. These factors lead to frustration, miscommunication and ultimately, the avoidance of necessary rehabilitation. To address these issues, it is crucial to integrate South African sign language into healthcare professional training, provide ongoing in-service education and develop more inclusive disability policies. These measures would contribute to reducing healthcare disparities and ensuring that patients with deafness have equal access to physiotherapy services, aligning with broader goals of universal health coverage and human rights.
